# Analyzing Fusion Pore Dynamics and Counting the Number of Acetylcholine Molecules Released by Exocytosis

**DOI:** 10.1021/jacs.4c08450

**Published:** 2024-09-11

**Authors:** Yuanmo Wang, Ajay Pradhan, Pankaj Gupta, Jörg Hanrieder, Henrik Zetterberg, Ann-Sofie Cans

**Affiliations:** † Department of Chemistry and Chemical Engineering, Chalmers University of Technology, Kemigården 4, SE-412 96 Gothenburg, Sweden; ‡ Department of Psychiatry and Neurochemistry, Institute of Neuroscience & Physiology, The Sahlgrenska Academy at the University of Gothenburg, SE-43141 Mölndal, Sweden; ⊥ Department of Neurodegenerative Disease, UCL Institute of Neurology, Queen Square, WC1N 3BG London, U.K.; ∥ Clinical Neurochemistry Laboratory, The Sahlgrenska University Hospital, SE-43141 Mölndal, Sweden; # UK Dementia Research Institute at UCL, WC1N 3BG London, U.K.; § Hong Kong Center for Neurodegenerative Diseases, Clear Water Bay, 999077 Hong Kong, China; ° Wisconsin Alzheimer’s Disease Research Center, University of Wisconsin School of Medicine and Public Health, University of Wisconsin−Madison, Madison, Wisconsin 53792, United States

## Abstract

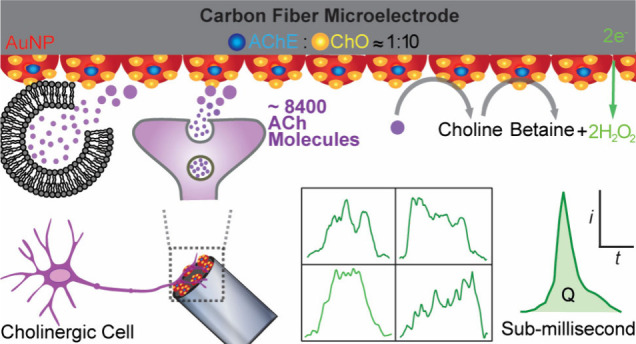

Acetylcholine (ACh) is a critical neurotransmitter influencing various neurophysiological functions. Despite its significance, quantitative methods with adequate spatiotemporal resolution for recording a single exocytotic ACh efflux are lacking. In this study, we introduce an ultrafast amperometric ACh biosensor that enables 50 kHz electrochemical recording of spontaneous single exocytosis events at axon terminals of differentiated cholinergic human SH-SY5Y neuroblastoma cells with sub-millisecond temporal resolution. Characterization of the recorded amperometric traces revealed seven distinct current spike types, each displaying variations in shape, time scale, and ACh quantities released. This finding suggests that exocytotic release is governed by complex fusion pore dynamics in these cells. The absolute number of ACh molecules released during exocytosis was quantified by calibrating the sensor through the electroanalysis of liposomes preloaded with varying ACh concentrations. Notably, the largest quantal release involving approximately 8000 ACh molecules likely represents full exocytosis, while a smaller release of 5000 ACh molecules may indicate partial exocytosis. Following a local administration of bafilomycin A1, a V-ATPase inhibitor, the cholinergic cells exhibited both a larger quantity of ACh released and a higher frequency of exocytosis events. Therefore, this ACh sensor provides a means to monitor minute amounts of ACh and investigate regulatory release mechanisms at the single-cell level, which is vital for understanding healthy brain function and pathologies and optimizing drug treatment for disorders.

Neuronal communication occurs through the rapid, calcium-triggered release of neurotransmitters from synaptic vesicles fusing with the cell plasma membrane.^1^ This process, known as exocytosis, happens in less than a millisecond,^2^ allowing neurotransmitters released to bind to receptors at neighboring neurons and transmit chemical signals.^1^ The amount and frequency of neurotransmitter released are important factors in determining the communication strength between neurons and shaping synaptic plasticity. This process underlies fundamental brain functions such as learning and memory and is also involved in the pathophysiology of brain disorders such as drug abuse and addiction, which are not yet fully understood.^3,4^ Acetylcholine (ACh) is a key excitatory neurotransmitter in the central and peripheral nervous systems (CNS and PNS),^5^ essential for regulating heart rate, controlling muscle contraction, and contributing to cognitive function.^6,5^ It also serves as a neuromodulator of glutamatergic and GABAergic synapses in the CNS.^7^ The impairment of the cholinergic system has been associated with several neurological and psychiatric disorders, such as Alzheimer’s disease and schizophrenia.^8^ Investigations of cholinergic neurotransmission and its role in complex cognitive behavior have led to the development of various analytical techniques for studying ACh signaling, such as *in vivo* microdialysis, capillary electrophoresis, mass spectrometry, neuroimaging, and photoelectrochemical biosensing.^9−11^ However, for an in-depth understanding of the regulatory aspects of ACh neurotransmission at synapses, methods that can spatially and temporally capture the dynamics of rapid synaptic vesicle ACh release during neuronal activity are needed.

Synaptic neurotransmission can be studied using electrochemical methods, where amperometry offers a straightforward application and superior temporal resolution (microseconds). By placing carbon fiber micro/nanoelectrodes at release sites of neuronal cells, redox current spikes resulting from the detection of neurotransmitters released during single exocytosis events can be recorded. This method enables real-time recording of vesicle fusion pore-controlled release dynamics of individual exocytosis events occurring on a sub-millisecond time scale.^12^ Further, amperometry provides a quantitative measure of synaptic signaling by converting the integrated total charge (*Q*) detected from current spikes and using Faraday’s law to calculate the number of molecules released during exocytosis.^12^ However, this technique is limited to electroactive neurotransmitters such as catecholamines and serotonin, which can be readily oxidized at the electrode surface. For non-electroactive neurotransmitters like ACh and glutamate, redox reactions at the carbon surface are not feasible. To overcome this limitation, a chemically selective biosensor can be created by modifying the electrode surface with enzymes that convert non-electroactive neurotransmitters into detectable reporter molecules, typically hydrogen peroxide (H_2_O_2_). While these biosensors are sensitive and selective, their detection capabilities have remained limited to subsecond time resolutions, which is too slow for monitoring individual exocytosis events.^13^ Insufficient technology for direct quantitative measurements of ACh has led to varying estimates of ACh vesicle quantal size, ranging from 400 to 22,000 ACh molecules, depending on the biological model system and analysis techniques used.^14−21^ To overcome this challenge, our lab introduced a novel biosensor design concept that improves the sensor speed by limiting enzyme coatings to a molecular monolayer. This approach minimizes diffusion distances for enzymatic electroactive reporter molecules to reach the electrode surface for detection.^22^ Compared to traditional sensors with thick enzyme layers, our minimal enzyme-coating concept has reduced response times by up to 3 orders of magnitude. With a 33 μm carbon fiber biosensor, we have captured ACh release from artificial cells in tens of milliseconds,^22^ and glutamate from exocytotic release in brain tissue and isolated synaptic vesicles in sub-milliseconds.^23,24^

In this work, we tailored our ultrafast sensor concept for single-cell ACh presynaptic measurements by immobilizing the sequential enzymes acetylcholinesterase (AChE) and choline oxidase (ChO) onto a 5 μm carbon fiber disc electrode surface (Figure 1A), providing dimensions for precise positioning against single-cell structures. To functionalize the carbon microelectrode surface, maximizing its sensing surface area, enhancing detection efficiency of H_2_O_2_, and providing a scaffold for the ultrathin enzyme coating, a dense layer of gold nanoparticle (AuNP) hemispheres, approximately 80 nm in diameter, was electrodeposited, creating a surface topology resembling that of cauliflower (Figure 1D, Figure S2). Enzymes AChE and ChO were immobilized onto the AuNP-modified sensor surface at a molar ratio of 1:10, as determined by a previous study optimizing sequential enzymatic catalysis of ACh (Figure 1A).^25^ The ACh biosensor created was placed in contact with axon terminals of differentiated cholinergic human SH-SY5Y neuroblastoma cells prepared using a well-characterized cholinergic protocol (Figure 1B,F and Figure S4).^26^ To minimize the risk of detecting catecholamines released from these cells, a constant potential of −0.5 V versus a Ag/AgCl reference electrode was applied to the ACh sensor surface.^22,23^ At this potential, the ACh sensor gained chemical selectivity against dopamine (Figure S3). Using a 50 kHz amperometric recording, the sensor successfully captured spontaneous bursts of isolated reduction current transients corresponding to individual ACh exocytotic events (*n* = 18 cells) at approximately at 28 ± 5 Hz (*n* = 16 cells), with the error representing standard error of the mean (SEM). The average current spike from these recordings resolved at the sub-millisecond time scale shows the typical shape characteristics of fusion pore regulated exocytosis with a rapid *T*_rise_ and slower *T*_fall_ (Figure 1E).

**Figure 1:**
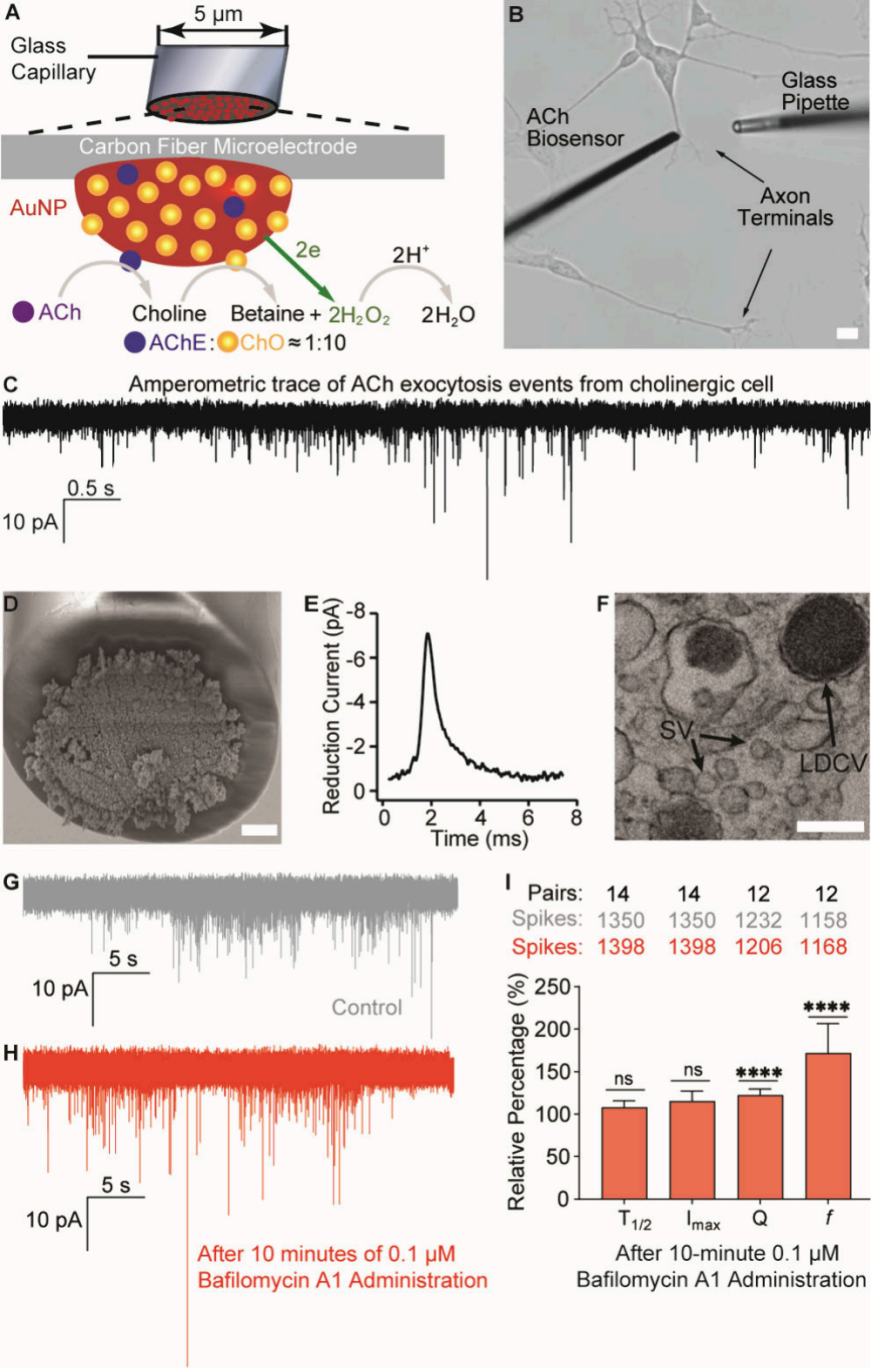
(A) Detection principle for the ultrafast ACh biosensor, modified with AuNPs (red hemispheres) and the enzymes AChE (blue) and ChO (yellow). Schematic not to scale. (B) Experimental setup with placing an ACh biosensor at an axon terminal of a differentiated cholinergic SH-SY5Y cell, opposing a glass pipet delivering stimulants or drugs. Scale bar is 10 μm. (C) A 50 kHz amperometry recorded current vs time trace of ACh exocytosis activity at an axon terminal. (D) Scanning electron microscope image of a 5 μm carbon electrode coated with a dense layer of ∼80 nm AuNPs. Scale bar: 1 μm. (E) An average amperometric current spike representing ACh release from single exocytosis events (*n* = 162). (F) Transmission electron microscope image of a differentiated cholinergic SH-SY5Y cell axon displaying the presence of ∼40 nm small clear core synaptic-like vesicles (SV) alongside large dense core vesicles (LDCV). Scale bar: 150 nm. (G) Exocytosis amperometry recording performed as a control experiment in the presence of 0.002% DMSO (gray) and (H) following a 10 min local administration of 0.1 μM bafilomycin A1 (red) using the same ACh biosensor at a precise target cell location. (I) Comparison of drug effect to single exocytosis events regarding ACh current spike halftime (*T*_1/2_), maximum amplitude (*I*_max_), total charge (*Q*), and frequency (*f*) (average of means ± standard error of the mean, SEM) with control. The two-tailed paired Mann–Whitney test was used, ****p < 0.0001.

To demonstrate how the ACh biosensor can be used to investigate the drug effect on ACh signaling, single cholinergic cells were exposed to 0.1 μM bafilomycin A1 (Figure 1G,H), a V-ATPase inhibitor known to also activate release of intracellular calcium stores, potentially affecting fusion pore dynamics and exocytosis activity.^27^ By recording ACh release from a single release site before and after a 10 min local administration of the drug using a glass microinjection pipet, an increase in both the exocytosis activity (170%) and quantal release (120%) was observed (Figure 1I, Figure S9 and Table S2). This finding is consistent with a previous study on catecholamine signaling in chromaffin cells and demonstrates the applicability of this technology for presynaptic studies.^28^

The high temporal resolution of our recordings (50 kHz) enabled the detection of a diversity of shapes and time courses for individual current spikes. Analyzing all spikes, we identified six spike types that were found similar in shape and frequency to those previously reported in brain tissue recordings of octopamine in *Drosophila melanogaster* and glutamate in rodents.^29,30^ Out of the spikes with a simple sharp shape, we observed two distinct populations based on their height: shorter (sharp) and taller (full sharp). The full sharp spikes presented here exhibited approximately twice the current amplitude (*I*_max_) of other spikes (Figure S6E) and have not been characterized in previous studies. Since the full sharp spikes exhibited the same integrated total charge (*Q*) as the increasing, decreasing, and plateau spikes, events previously associated with full release, we designated it as “full sharp”.^23^Figure 2D illustrates the identified spike shapes showing that “sharp spikes” have the highest abundance of all spikes (42%) and full sharp have a 9% prevalence. This spike heterogeneity indicates that the fusion pore is highly regulated by factors controlling its opening and closing and leads to presynaptic tuning of ACh release in these cells.

**Figure 2:**
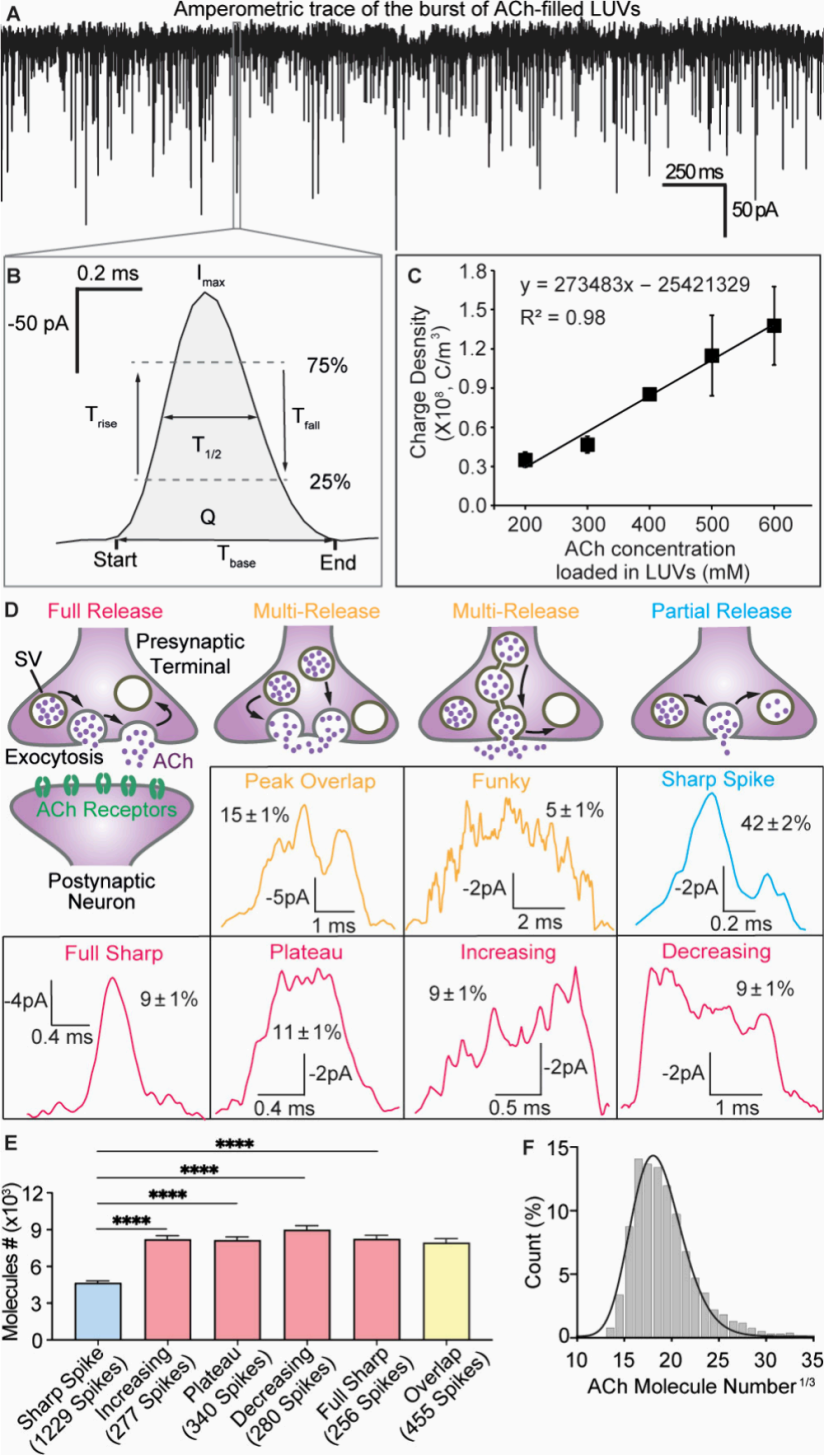
(A) An amperometric trace showing reduction current transients detecting ACh release from LUVs filled with 400 mM ACh that rupture stochastically at the sensor surface under a −0.5 V potential versus a Ag/AgCl reference. (B) Each current transient from single release events provides kinetic and quantitative information. (C) Calibration curve plotting the mean charge density (average ± SEM) from LUV electroanalysis vs LUV preloaded ACh concentration (*n* = 4–5 LUV samples, 600–2000 spikes/ACh concentration). (D) Relating the ACh quantal release from single exocytosis events to the various detected current spike shapes and their abundance (%). (E) Comparison of ACh quantal release vs different category of current spike shapes (average of means ± SEM) using two-tailed unpaired Student’s *t* test, ****p < 0.0001. (F) LogNormal histogram (bin size 1, *R*^2^ = 0.98) displaying the cubic root transformed distribution of ACh molecules released from single exocytosis events. (D–F) Data are collected from 18 cells, and 2945 amperometric current spikes are recorded.

To accurately quantify the number of ACh molecules by exocytotic release, an appropriate calibration of the sensor signal is needed. Here we used a liposome-based calibration strategy, pioneered by our lab for quantifying glutamate.^31^ This method involves immersing the ACh sensor into a solution of extruded large unilamellar liposomes (LUVs, ∼180 nm in diameter, Figure S10A) preloaded with various ACh concentrations (200 to 600 mM) and performing amperometric electroanalysis of single LUV content.^32,33^ By applying a potential of −0.5 V versus a Ag/AgCl reference to the biosensor, individual LUVs in contact with the sensor surface stochastically rupture, and the ACh content released is detected as current spikes. Figure 2A and B display a typical redox current time trace, detecting ACh release from single LUVs prefilled with a 400 mM ACh solution, demonstrating detection of single current spikes on a sub-millisecond time scale (Figure S10B, Table S3). The measured *Q* from individual redox current spikes obtained through liposome electroanalysis (Figure 2B, Table S3) was used to calculate the average charge density (*Q*/LUV volume). A linear relationship between the charge density and encapsulated ACh concentration in LUVs established a calibration curve for cellular recordings (Figure 2C). This calibration curve was then used to quantify the absolute number of ACh molecules released during exocytosis for each spike type. Figure 2E illustrates that the average exocytosis release during the most prevalent, “sharp” events corresponds to an average of 4700 ± 200 ACh molecules (mean ± SEM), while plateau-shaped and full sharp events release 8400 ± 300 ACh molecules (mean ± SEM). Additionally, Figure 2F shows that pooling all release events detected into a histogram, a LogNormal equation fit estimates an average release of 5800 ACh molecules across all spike modes. This estimation aligns with the consensus of 5000 to 10,000 ACh molecules per vesicle.^34^ Assuming that the plateau-shaped spike types detected in these cholinergic cells correspond to full exocytosis, as suggested by previous glutamate measurements,^30,35^ and considering an internal vesicle solution volume of 20 zL,^36^ we estimate the original vesicular ACh concentration to be 0.7 ± 0.03 M. This concentration is consistent with findings regarding the storage of glutamate and catecholamine in secretory vesicles.^35,37,38^

In summary, we have developed an ultrafast ACh biosensor and demonstrated its application for monitoring the exocytotic release from presynaptic sites at human differentiated cholinergic cells. The sensor’s high temporal resolution facilitated the detailed study of fusion pore-controlled quantal release and its modulation by pharmacological treatment. This ultrafast technology provides a novel approach for investigating the regulatory mechanisms of ACh release and the impact of pharmacological treatment.

## Supporting Information Available

Experimental details and additional data on ACh biosensor characterization, images and amperometric exocytosis recording at the differentiated human cholinergic cells, with and without drug administration, and electroanalysis of ACh-filled LUVs, including Figures S1–S10 and Tables S1–S3 (PDF)
