# Supplementary material for: Analyzing Fusion Pore Dynamics and Counting the Number of Acetylcholine Molecules Released by Exocytosis
Source: J Am Chem Soc. 2024 Sep 11;146(38):25902–6. doi: 10.1021/jacs.4c08450 (PMC11440489; doi:10.1021/jacs.4c08450)
Supplement: Supplementary file 1 — ja4c08450_si_001.pdf [file ja4c08450_si_001.pdf]

Analyzing Fusion Pore Dynamics and Counting the Number of Acetylcholine Molecules Released by Exocytosis

Yuanmo Wang,[†] Ajay Pradhan,[‡] Pankaj Gupta,[†] Jörg Hanrieder,[‡,⊥] Henrik Zetterberg,[‡,⊥,∥,#, §,°] Ann-Sofie Cans*[,†]

[†]Department of Chemistry and Chemical Engineering, Chalmers University of Technology, Kemigården 4, SE-412 96 Gothenburg, Sweden

[‡]Department of Psychiatry and Neurochemistry, Institute of Neuroscience & Physiology, the Sahlgrenska Academy at the University of Gothenburg, SE-43141 Mölndal, Sweden

[⊥]Department of Neurodegenerative Disease, UCL Institute of Neurology, Queen Square, WC1N 3BG London, UK

[∥]Clinical Neurochemistry Laboratory, Sahlgrenska University Hospital, SE-43141 Mölndal, Sweden

[#]UK Dementia Research Institute at UCL, WC1N 3BG London, UK

[§]Hong Kong Center for Neurodegenerative Diseases, Clear Water Bay, 999077 Hong Kong, China

[°] Wisconsin Alzheimer's Disease Research Center, University of Wisconsin School of Medicine and Public Health, University of Wisconsin-Madison, Madison, WI 53792, USA

Corresponding author email address:

* cans@chalmers.se

Table of Content

I. Experimental Procedure……………………………………………………………………………………S2
II. Additional Data…………………………………………………………………….....................................S7
III. References………………………………………………………………………….................................S15

I. Experimental Procedure

Materials. Acetylcholine chloride (AChCl), 10 KU acetylcholinesterase (AChE) from *Electrophorus electricus*, 500 UN choline oxidase (ChO) from *Alcaligenes sp.*, sodium bicarbonate ($NaHCO_3$), gold chloride trihydrate ($HAuCl_4$), ferrocene methanol (FcMeOH), sulfuric acid (H_2SO_4), copper sulfate ($CuSO_4$), Whatman® Anotop® 25 syringe filters with 20 nm filtering size, glucose, sodium chloride (NaCl), fetal bovine serum (FBS), retinoic acid (RA), dimethyl sulfoxide (DMSO), FM™ 1-43 dye, poly-L-ornithine and osmium tetroxide (OsO_4) were purchased from Sigma-Aldrich (St. Louis, MO, USA). HEPES, magnesium chloride ($MgCl_2$), potassium chloride (KCl) and DMEM/F12 with Glutamax™ were purchased from Fisher Scientific (Pittsburgh, PA, USA). 1,2-dioleoyl-sn-glycero-3-phosphocholine (DOPC), 1,2-dioleoyl-sn-glycero-3-phosphoethanolamine (DOPE) and cholesterol were purchased from Avanti Polar Lipids, Inc. (Birmingham, AL, USA). Glutaraldehyde, formaldehyde, gelatin capsules and copper Gilder TEM Finder Grids were purchased from Electron Microscopy Sciences (Hatfield, PA, USA). Calcium chloride ($CaCl_2$) was purchased from Honeywell (Charlotte, NC, USA). All solutions were prepared in MilliQ water with resistivity ≥ 18 MΩ.cm.

Fabrication of 5 µm carbon fiber microelectrodes (CFME). The acetylcholine (ACh) biosensors described here are surface modified 5 µm disc CFME. These CFME were first produced using a modified version of the previously described protocol for 33 µm disc CFME.[1,2] In brief, single 5 µm carbon fibers were first aspirated into a borosilicate glass capillary (1.0 mm outer diameter, 0.5 mm inner diameter, Sutter Instruments, Novato, CA, USA). The borosilicate capillaries with the carbon fiber protruding in both ends were then placed in the holder of a heat-filament micropipette puller (Model P-100, Sutter Instruments, Novato, CA, USA) and by applying heat and pulling force onto the glass capillaries, two tapered glass insulated cylindrical carbon fiber tip electrodes were produced. To ensure no gap space between the surrounding glass and the carbon fiber that may cause leakage, the electrode tips were placed into epoxy resin (EpoTek 301, Epoxy Technology, Billerica, MA, USA) for 3 minutes and 15 seconds dip in acetone to remove excess epoxy, following by overnight curing of the epoxy in an oven at 100 °C. The cured electrodes were then placed under a Nikon TMS inverted microscope (Nikon, Tokyo, Japan), and precise cuts were made near the carbon fiber-glass junction with a sharp scalpel or cutting blade. To obtain a smooth, flat surface at the electrode tip, the electrodes were bevelled at an angle close to vertical, approximately 80 degrees, using a micropipette-mounted beveller (model BV-10, Sutter Instrument Co., Novato, CA, USA), producing electrodes insulated with glass and having an exposed smooth disc electrode surface. The electrodes were backfilled with 3 M KCl solution, with insertion of a silver wire for connection to the potentiostat.

Testing CFME using cyclic voltammetry. To test the electrode performance, cyclic voltammetry was conducted in 1 mM FcMeOH solution using a scanning voltage ranging from -0.2 V to +0.8 V against a Ag/AgCl reference electrode (CH Instruments, Inc. Austin, TX, USA) at a scan rate of 0.1 Vs^{-1}. Only electrodes showing stable steady-state currents with reasonable current amplitudes were chosen for further surface modification and functionalization.

Surface modification of CFMEs, creating ultrafast amperometric ACh biosensors. Here, to fabricate an ultrafast ACh biosensors a method previous described,[3] was used with modifications. Briefly, the surface of CFMEs was modified with gold nanoparticles (AuNP) and an ultrathin layer of the sequential enzymes, acetylcholine esterase (AChE) and choline oxidase (ChO). To provide a conductive scaffold for the enzymes to immobilize at the electrode surface and a material suitable for the detection of the enzymatic catalysis product, hydrogen peroxide (H_2O_2), gold nanoparticle (AuNP) hemispheres were electrodeposited at the CFME surface. This method used was however altered to create a higher AuNP density at the electrode surface for enzymes to bind and to enhance the biosensor sensitivity. Briefly, a freshly bevelled 5 µm diameter carbon fiber disc microelectrode and a Ag/AgCl reference electrode were connected to a potentiostat (model 650A Series Multi-Potentiostat, CH Instruments, Inc. Austin, TX, USA), and immersed in a 0.5 mM $HAuCl_4$ solution dissolved in 0.5 M H_2SO_4. A potential of +1.2 V was applied to the carbon fiber electrode surface for 10 seconds, followed by a potential step to -0.6 V for 24 seconds, resulting in the formation of AuNPs with an average size of 80 nm (Figure S2). To estimate the total surface area of AuNP deposited onto the CFME surface, a modified method to Finot et. al[4] was used. Here the AuNP coated CFME surface area was placed into 0.5 M H_2SO_4 and a potential of +1.4 V was applied to the CFME surface against a Cu/$CuSO_4$ reference electrode for 5 s. This was followed by a linear sweep from +1.4 V to +0.4 V at a scan rate of 0.1 Vs^{-1} in 500 mM H_2SO_4. To determine the total charge of the induced redox reaction that occurred at the AuNP surface, the reduction peak recorded at approximately +0.8 V was integrated and the surface area of the Au NP can be calculated using the previously reported constant of 489 µC cm^{-2}.[4]

The final step was to coat an ultrathin layer of enzymes onto the surface of AuNP-coated CFMEs using a previously developed protocol from our lab.[5] Briefly, an enzyme solution containing AChE and ChO was freshly prepared in 50 mM sodium phosphate buffer (pH 7.4). An optimal molar ratio of 1:10 for AChE:ChO was used to obtain a maximum sequential enzymatic catalysis efficiency of the enzymes after immobilization. The tip of the CFME was immersed in 200 µL of enzyme solution for approximately 2-3 hours at room temperature in the dark. This resulted in the

immobilization of an ultrathin layer of enzymes onto the AuNP coated surface through enzyme self-adsorption creating functional ACh biosensors. The freshly prepared ACh biosensors were then either used within 6 hours for experiments or stored in 10 mM phosphate buffered saline (PBS, pH=7.4) at 4 °C up to one day. Only ACh biosensors that have undergone proper quality control were utilized for subsequent amperometric measurements to ensure accurate and reliable results. The quality test of ACh biosensors included checking for baseline stability, low background noise and absence of abnormal current transients when placing the sensor in the isotonic cell recording solution without touching the cells.

Characterization of AuNP-coated microelectrode surface. To characterize the AuNP size, particle coverage and surface topology, scanning electron microscopy (SEM; JEOL JSM-7800F, JEOL GmbH, Freising, Germany) was used for imaging the AuNP modified CFME surface. To prepare electrodes for SEM imaging, each microelectrode was backfilled with silver paste coated silver wire to connect with the carbon fiber on the inside of the glass capillary. After the paste was dried, the electrodes were firmly grounded to the SEM sample stage through the silver wire and conductive carbon tape (Ted Pella Inc, Redding, CA, USA) to minimize charging effects during SEM imaging.

Electrochemical characterization of the acetylcholine biosensor's responses to acetylcholine and dopamine. Cyclic voltammetry and differential pulse voltammetry were used to perform a chemical selectivity test of the ACh biosensor and was tested against the potential chemical interferent, dopamine, a neurotransmitter that may also be secreted by differentiated cholinergic SH-SY5Y cells. The voltametric measurements were performed using a potentiostat (Model 1030C Multi-channel Potentiostat, CH Instruments, Inc., Austin, TX, USA). For cyclic voltammetric measurements, the potential was swept from 0 V to -0.6 V versus a Ag/AgCl reference electrode and then returned, at a scan rate of 0.1 V/s. The biosensor's response was initially tested in a bulk solution of 10 mM PBS (pH 7.4), followed by exposure to increasing concentrations of acetylcholine solutions, ranging from 0.01 mM to 0.25 mM. After washing the sensor tip in MilliQ water, the same sensor was subsequently tested in dopamine solutions at 0.25 mM (Sigma Aldrich, St. Louis, MO, USA). Both acetylcholine and dopamine solutions were prepared in 10 mM PBS buffer (pH 7.4). Differential pulse voltammetry, a more sensitive voltametric technique, was then applied to acetylcholine solutions, ranging from 0.01 mM to 0.5 mM, which were found to fall within the sensor´s linear range. The same biosensor was subsequently tested in dopamine solutions at 0.25 mM and 0.5 mM after being washed by MilliQ water. The differential pulse voltammetry settings were optimized using a 5 µm disc carbon electrode in a 1 mM FcMeOH solution. The voltammetric measurements were performed using an initial potential of 0 V, a final potential of -0.8 V, and a potential increment of 0.004 V. The pulse width and amplitude were 1 second and 0.1 V, respectively. Additionally, the sampling width was set to 0.02 seconds, and the pulse period to 0.5 seconds. All measurements were conducted at room temperature in a Faraday cage after purging with nitrogen gas.

Differentiation of human cholinergic cells. SH-SY5Y human neuroblastoma cells (ECACC 94030304) were cultured in DMEM/F12 with Glutamax^{TM} supplemented with 10 % fetal bovine serum (FBS), with media replacement every two days. The cells were incubated at 37 °C with 5 % CO_2 in a humid environment and were passaged upon reaching 80 % confluency. To obtain cholinergic cells that release ACh for amperometric measurement using the ACh biosensor, the SH-SY5Y human neuroblastoma cells were differentiated into a cholinergic phenotype using the method described by de Medeiros et al. with minor changes (Figure S1).[6] In brief, undifferentiated SH-SY5Y neuroblastoma cells were seeded onto 35 mm MatTek dishes with glass coverslip bottoms (MatTek, Ashland, MA, USA) coated with poly-L-ornithine and Biolaminin 521 (BioLamina AB, Sundbyberg, Sweden) at a density of 5000 cells/cm^2 in DMEM/F12 medium containing 10 % FBS. The following day, the medium was replaced with DMEM/F12 containing 1 % FBS and 10 µM retinoic acid (RA) to initiate cholinergic differentiation. After four days of pre-treatment with RA, the medium was changed to DMEM/F12 supplemented with 1 % FBS, 10 µM RA, and 50 ng/mL brain-derived neurotrophic factor (BDNF, PrepoTech, Cranberry, NJ, USA). The cells were allowed to differentiate for an additional 4-5 days (Days 8-9) prior to amperometric measurements and imaging.

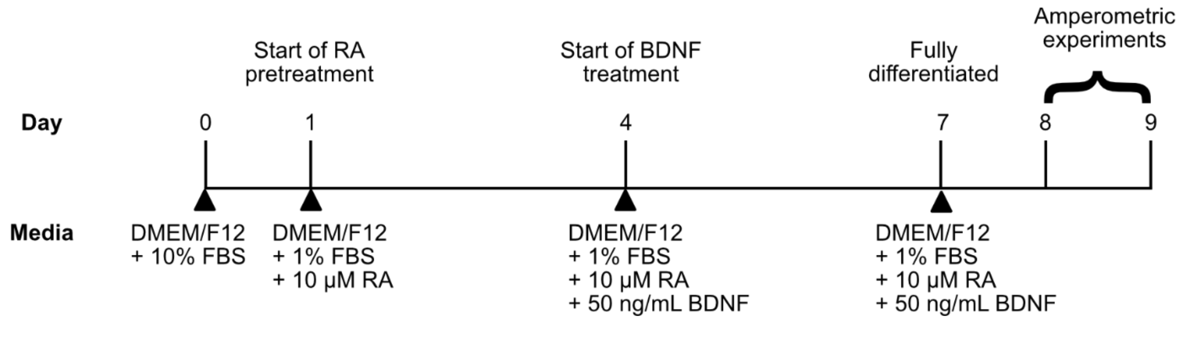

Figure S1. Schematic illustration of the cholinergic differentiation of the SH-SY5Y human neuroblastoma cell line. The cells were differentiated into a cholinergic phenotype for seven days using RA and BDNF.

High-resolution imaging of cholinergic cells using transmission electron microscope (TEM). SH-SY5Y human neuroblastoma cells were cultured at a seeding density of 50000 cells/cm^2 and differentiated into cholinergic cells using RA and BDNF as described earlier. On the seventh day of differentiation, the mature cholinergic cells were gently washed with prewarmed 0.1 M sodium cacodylate buffer (Agar Scientific, Stansted, UK; 37 °C), followed by a 30-minute incubation in Karnovsky's fixative containing 0.05 M cacodylate buffer (pH=7.4) with the additives 2.5% glutaraldehyde, 2% formaldehyde and 0.02% sodium azide (BDH Chemicals, Poole, UK) at room temperature in the dark. The fixative was rinsed away five times with five-minute washes using 0.1 M sodium cacodylate buffer. To stain lipid membranes, the culture dish was then incubated for 30 minutes in 1% OsO_4 at room temperature in the dark and with caution when handling. The OsO_4 was removed with three five-minute washes in distilled water. The cells were further stained with uranyl acetate (Merck, Darmstadt, Germany), by incubating the cells with 1% uranyl acetate for 30 min at room temperature before washing the staining agent away using distilled water. The cells were progressively dehydrated using ethanol (Fisher Scientific, Loughborough, UK) concentrations ranging from 30% to 100%, with an incubation time of five minutes per concentration. The dehydrated cells were then infiltrated with increasingly higher concentrations of epoxy resin (Hard Plus Resin 812 Kit, EMS, Hatfield, PA, USA), ranging from 25% to 100%, for 20 minutes per concentration. After 60 minutes in 100% resin, the cells were embedded in gelatin capsules copper Gilder TEM Finder Grids and incubated at 60 °C overnight for curing. The capsules were cut at a thickness of 80 nm and applied onto copper Gilder TEM Finder Grids (Electron Microscope Sciences, Hatfield, PA, USA) and imaged using a FEI Talos L120C TEM (Field Electron and Ion Company, Hillsboro, OR, USA).

Localization of small clear core synaptic-like vesicles (SVs) in cholinergic cells for amperometric measurements. To visualize the location of intracellular SVs and determine the best sites for positioning of the ACh biosensor tip for amperometric measurements, the styryl dye FM1-43 (Invitrogen, UK), which can label the lipid bilayer of biomembranes,[7] was used. The FM1-43 dye was internalized via the compensatory endocytosis events during the vesicle recycling process by stimulating the neuronal cells to undergo exocytosis in the presence of the dye added in the extracellular media.[8] The differentiated cholinergic cells with an initial seeding density of 5000 cells/cm^2 were first incubated for 7 minutes at 37 °C in serum-free DMEM/F12 containing 10 μM FM1-43, followed by a 20-minute stimulation in a serum-free DMEM/F12 solution consisting of 56 mM KCl and 10 μM FM1-43 at 37 °C. After a thorough five-time rinse using isotonic HEPES buffer (10 mM HEPES, 2 mM $CaCl_2$, 1.2 mM $MgCl_2$, 5 mM glucose, 150 mM NaCl, 5 mM KCl, pH 7.4), the cells were placed on a microscope heating stage (Bioscience Tools, Highland, USA) to maintain a temperature of approximately 31 °C for imaging using Leica DM IRB inverted microscope (Leica Microsystems, GmbH, Wetzler, Germany) in fluorescence mode with a 40X objective lens.

Amperometric measurements of ACh exocytosis events in differentiated human cholinergic cells. By using a freshly prepared ACh biosensor, amperometric measurements of ACh exocytosis release from differentiated human cholinergic cells were performed from day 8 to day 9 of the differentiation. The cells cultured in 35 mm MaTek dishes were first rinsed three times with pre-warmed isotonic HEPES buffer (10 mM HEPES, 2 mM $CaCl_2$, 1.2 mM $MgCl_2$, 5 mM glucose, 150 mM NaCl, 5 mM KCl, pH 7.4). Approximately 2 mL of the isotonic HEPES buffer solution was added to the dish for measurement, with periodic addition upon evaporation. The dish containing cells was placed on the microscope stage of the Leica DM IRB inverted microscope (Leica Microsystems, Wetzlar, Germany) fitted with a stage heater (Bioscience Tools, San Diego, CA, USA) to maintain a temperature of approximately 31 °C at the dish region containing the cells. To precisely place the ACh biosensor onto a specific cell structure, the biosensor was positioned in close contact to the axon terminal of the cell with the aid of a micromanipulator (Scientifica, East Sussex, UK) under microscope equipped with objective lens ranging from 10 X to 40 X. A constant potential of - 0.5 V *versus* a Ag/AgCl reference electrode was applied onto the sensor surface and the amperometric time traces of ACh release from the cells were recorded at 50 kHz. A lowpass Bessel filter of 1 kHz was used via an internal filter. All measurements were conducted with the heating element off, inside a Faraday cage to prevent external noise, using ACh biosensors with stable baselines and no aberrant noise.

Investigation of the effect of the drug bafilomycin A1 on ACh exocytosis release. To investigate the acute effects of 0.1 μM bafilomycin A1 (Cell Signaling Technology, Danvers, MA, USA) on cholinergic exocytosis, single cell amperometric measurements were further performed on the differentiated human cholinergic cells at day 8 and day 9 of differentiation. Since bafilomycin A1 is insoluble in water, stock bafilomycin A1 was first prepared in 100 % (v/v) DMSO followed by subsequent dilutions to result in a final DMSO concentration of 0.002 % (v/v). A borosilicate glass capillary (1.0 mm outer diameter, 0.5 mm inner diameter, Sutter instrument, Novato, CA, USA) was pulled into a sharp tip using a micropipette puller (Model P-100, Sutter Instruments, Novato, CA, USA), and beveled to result in an opening diameter

of approximately 5 µm and attached to a FemtoJet microinjector (Eppendorf, Hamburg, Germany) after filling it with 0.1 µM of bafilomycin A1 dissolved in isotonic HEPES buffer containing 0.002 % DMSO.

Before amperometric measurements, the differentiated cells cultured in a 35 mm MatTek dish were rinsed three times using pre-heated isotonic HEPES buffer (10 mM HEPES, 2 mM $CaCl_2$, 1.2 mM $MgCl_2$, 5 mM glucose, 150 mM NaCl, 5 mM KCl, pH 7.4). The culture dish was then filled with 2 mL of the isotonic HEPES buffer supplemented with 0.002 % (v/v) DMSO for control measurements. The micropipette loaded with bafilomycin A1 was positioned aside of the targeted cell while keeping a distance of about 50 µm away during entire measurements to avoid creating mechanical noise by the pipette and interference caused by drug solution exchange. After positioning the biosensor onto the axon terminal, the control amperometric measurements were initiated by applying a constant potential of -0.5 V against to the ACh sensor surface versus a Ag/AgCl reference electrode and performing a 100 kHz amperometric recording for approximately 3 minutes. Using the drug-filled glass micropipette, bafilomycin A1 solution was then locally sprayed at a target cell continuously for 10 min by applying a constant 600 hPa pressure from the FemtoJet, while performing a continuous amperometric recording throughout the time of drug delivery and until 3 min after the drug administration was terminated. The cell measurements were performed at approximately 31 °C inside a Faraday cage, using a 100 kHz recording speed and a lowpass Bessel filter of 1 kHz was applied via an internal filter built in the electrochemical instrument.

Preparation of large unilamellar vesicles (LUV) filled with acetylcholine (ACh) solution. Lipid-based vesicles, filled inside with ACh solution, were prepared via the thin lipid rehydration method as previously described with minor alterations.[1] In brief, DOPC, DOPE and cholesterol dissolved in chloroform were first added into a round bottom flask in the mass ratio of 50:25:25 (molar ratio of 39:21:40). The flask was connected to a rotoevaporator (Rotavapor R-114, BUCHI Labortechnik GmbH, Essen, Germany) and dried under vacuum for 3 hours at 40 °C. After complete evaporation of chloroform and the formation of a thin lipid film at the walls of the round bottom flask, 10 mM HEPES buffer (pH 7.4) containing a physiologically relevant concentration of ACh was added into the round bottom flask to rehydrate the lipid film and facilitate lipid vesicle formation. The solution was thoroughly mixed and reattached to the rotoevaporator and allowed to rotate without vacuum at room temperature for 30 minutes. The flask was removed and subjected to a few seconds of vortexing to thoroughly separate the lipid film from the glass wall with a final lipid concentration at about 2.5 mg/ml. The liposome solution was then subjected to five cycles of freeze-thawing in liquid nitrogen and room temperature water respectively, to ensure a homogenous ACh solution inside the liposomes that is similar to the ACh bulk solution.[9] For the unification of liposome size, the liposome solution was then extruded 21 times through a 400 nm pore sized polycarbonate membrane (Whatman plc, Maidstone, U.K.) using an Avanti Mini-Extruder (Avanti Polar Lipids, Inc., Birmingham, AL, USA) under a uniform pressure of 1 bar generated by nitrogen gas at room temperature. To filter out unencapsulated ACh molecules in surrounding solution, the extruded LUVs were separated from the ACh bulk solution using size exclusion centrifugation columns (Amersham™ MicroSpin™ S-200 HR Columns, Cytiva, Marlborough, MA, USA). The LUV solution was then immediately diluted in isotonic 10 mM HEPES buffer (pH 7.4), that was osmotically balanced with the interior osmotic pressure of the LUVs using NaCl, prior to LUV size measurements and amperometric measurements of the LUV ACh content. The hydration solution for rehydrating dried lipid film was prepared by dissolving AChCl in 10 mM HEPES buffer pH 7.4 at five different concentrations: 200 mM with an osmotic pressure of 365 mOsm/kg, 300 mM with 555 mOsm/kg, 400 mM with 703 mOsm/kg, 500 mM with 900 mOsm/kg and 600 mM with 1035 mOsm/kg.

Size measurements of ACh-filled LUVs. For the measurement of size distribution of the ACh-filled LUVs, the NanoSight LM10 Nanoparticle Tracking Analysis system (NTA) (Malvern Instruments, Ltd, Malvern, UK) was applied as described previously.[1] Immediately after LUVs preparation, the LUV solution was initially diluted 2000-fold in an isotonic 10 mM HEPES buffer, which had been filtered through Whatman® Anotop® 25 syringe filters with pore size of 20 nm, and further dilution was made depending on the outcomes to achieve the optimal concentration for the LUV size measurement. Multiple rounds of repeated measurements were conducted at a constant temperature of 25 °C to capture the size and size variability of the LUVs.

Amperometric measurements of ACh content in ACh-filled lipid vesicles. The quantitative amperometric measurements of ACh in ACh-filled lipid vesicles were conducted with slight modifications following a previously described method (Wang, Fathali, et al., 2019).[1] Prior to amperometric measurements, the liposome solution was diluted 5000-fold in a 10 mM HEPES buffer solution containing NaCl with an osmotic pressure equivalent to the osmotic pressure inside the LUVs. To prevent any possibility of change in LUV size due to osmotic effects, the measurements were carried out on the same LUV stock sample and HEPES buffer used for NTA measurements in parallel. A freshly prepared ACh biosensor backfilled with 3 M KCl solution was connected to an Axopatch 200B amplifier (Molecular Devices, San Hose, CA, USA) with a low-noise Axon Digidata 1550B digitizer (Molecular Devices, San Hose, CA, USA) via a

headstage. The ACh biosensor was then carefully lowered into a 500 μL droplet of diluted liposome solution placed onto the surface of a 50 mm glass coverslip (Epredia, Breda, Netherlands). A constant holding potential of 0 V was first applied to the sensor for 10 seconds, followed by immediately switching the potential to -0.5 V *versus* a Ag/AgCl reference electrode to promote the initial stochastic bursting of liposomes at the sensor surface. The amperometric detection of ACh release from individual LUVs bursting at the sensor surface was recorded for approximately five minutes. All amperometric measurements were performed inside a Faraday cage at room temperature and sampled at 20 kHz with an internal lowpass Bessel filter of 1 kHz.

Analysis of amperometric data. The amperometric recordings were analyzed using IgorPro 6.37 and IgorPro 9 software (WaveMetrics, Lake Oswego, OR, USA) with the assistance of an Igor Procedure File developed by David Sulzer's lab for single amperometric current spike analysis.[10] Using the binomial smoothing filter, the traces were first smoothed to 5 kHz. Spike events with the amplitude of the current exceeding 5 times the standard deviation of the background noise was regarded as real signals. Each individual currents spike in all traces were manually examined in order to remove false-positive signals. Various current spike parameters were defined for analysis, including T_{rise} and T_{fall}, which represent the rise and fall time between 25% and 75% of a peak signal; $T_{1/2}$, which is the spike width at half of the maximum current amplitude; I_{max}, which is the highest current of a peak; and Q, which is the charge detected in each current peak by integrating the area under the current spike.

II. Additional Data

This section of the supportive information presents additional data related to the characterization of the fabricated amperometric ACh biosensor presented in this work, the establishment of a calibration curve based on electroanalysis of the ACh content in single ACh-filled liposomes and use of the ACh biosensor for *in vitro* recording of ACh release at single differentiated human cholinergic SH-SY5Y cells, which were used as a cell model system. Characterization of the differentiated human cholinergic cells were performed to analyze the abundance of clear core synaptic-like vesicles, presumably filled with ACh. Moreover, the data of drug effects on ACh exocytosis are described in detail. In summary, the additional data complements the study's results by establishing a reliable method for the quantification of ACh content in intravesicular compartments.

Characterization of the electrodeposited AuNP size at the CFME surface.

To characterize the electrodeposited AuNP coating on the ACh sensor surface, the diameter of electrodeposited AuNPs on the 5 µm disc CFMEs surface was measured. This was done by performing image analysis of scanning electron microscopy images of the AuNP-modified CFME surfaces (Figure S2 A) using Image J software.[11] Figure S2 B shows a resultant histogram representing the size distribution of AuNPs (n=1085) measured at the electrode surface Gaussian curve fitting of the AuNPs size distribution estimates the mean diameter of AuNPs was to be approximately 77 nm median diameter of around 81 nm.

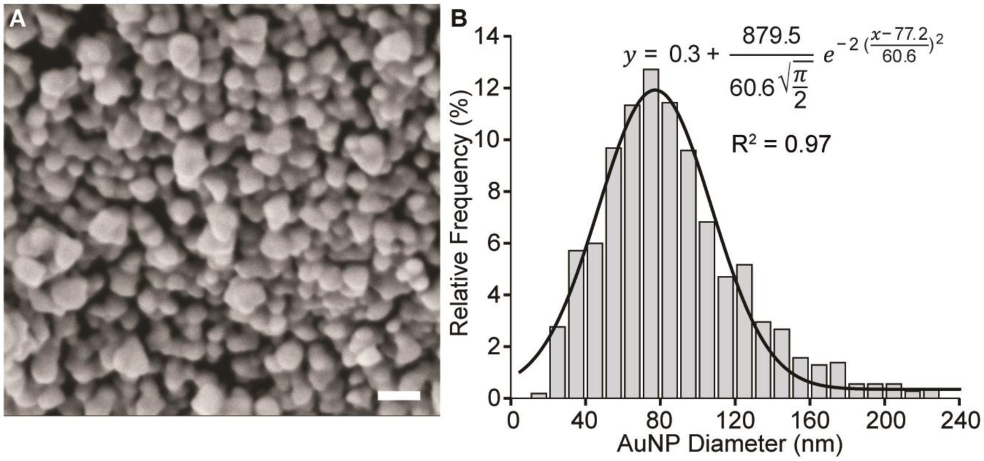

Figure S2. (A) Scanning electron microscopy image of gold nanoparticles (AuNPs) electrochemically coated onto a 5 µm disc shaped CFME. Scale bar is 100 nm. (B) Histogram of AuNP size distribution (n=1085) at the surface of a CFME as determined by image analysis, with a bin size of 10 nm and a correlation coefficient of 0.97.

Characterization of the acetylcholine biosensor chemical selectivity

The neurotransmitter phenotype of SH-SY5Y cells can potentially remain heterogeneous even after differentiation into a cholinergic phenotype, with the presence of dense core vesicles indicating the potential for catecholamine release. To assess the acetylcholine biosensor's specificity and response to potential interference from catecholamines secreted by differentiated cholinergic SH-SY5Y cells, the sensor´s electrochemical performance was tested in bulk solutions of increasing concentrations of acetylcholine and dopamine, using cyclic voltammetry and differential pulse voltammetry. Voltammograms generated from both voltametric techniques (Figure S3), indicate that the acetylcholine biosensor displayed good sensitivity to acetylcholine, but not to dopamine, at the reduction potential of -0.5 V versus a Ag/AgCl reference electrode. However, during cyclic voltammetry scans, the current generated by these sensors was relatively low and suspectable to tilting, likely due to non-faradaic effects, with only a modest increase in current with increasing ACh concentrations. In contrast, differential pulse voltammetry measurements, proved more sensitive and free from background non-faradaic effects, demonstrating a clear linear increase in sensor response with increasing acetylcholine concentrations at -0.5 V versus a Ag/AgCl reference electrode. These concentrations were carefully optimized to prevent enzyme saturation while still ensuring a sufficiently high signal output. In similar experiments conducted in 10 mM PBS, dopamine concentrations of 0.25 mM and 0.5 mM produced negligible responses at a reduction potential of -0.5 V versus a Ag/AgCl reference electrode. These findings suggest that the ACh biosensors used in this study demonstrate strong sensitivity to ACh at a negative reduction potential of -0.5 V, in contrast to dopamine, which is a potential major interferant, that may have been released from the differentiated cholinergic SH-SY5Y cells.

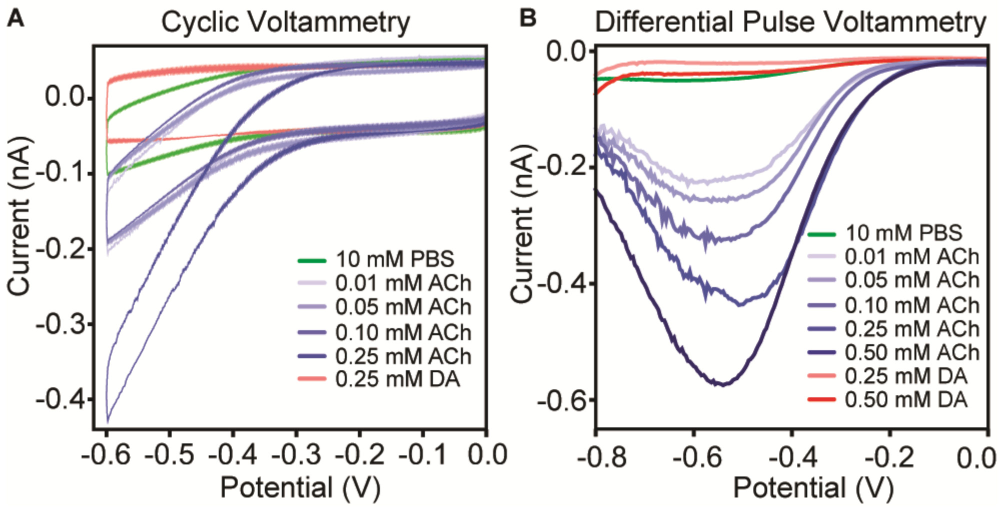

Figure S3. Response of an acetylcholine (ACh) biosensor to acetylcholine and dopamine (DA). (A) A representative cyclic voltammogram illustrating the response of an ACh biosensor to ACh solutions with concentrations ranging from 0.01 mM to 0.25 mM (blue gradient, with lighter for lower concentrations and darker for higher concentrations), as well as to DA solutions at concentrations of 0.25 mM (light red). A 10 mM PBS (green) served as a blank control. The potential was scanned from 0 V to -0.6 V against a Ag/AgCl reference electrode at a scan rate of 0.1 V/s. (B) Differential pulse voltammograms (from 0 V to -0.8 V against a Ag/AgCl reference electrode) displaying the response of the same ACh biosensor to increasing concentrations of ACh (0.01 mM to 0.5 mM, blue gradient, with lighter for lower concentrations and darker for higher concentrations) and DA (0.25 mM, lighter red, and 0.5 mM, dark red) with 10 mM PBS (green) as the blank control. In both experiments, acetylcholine and dopamine solutions were prepared in 10 mM PBS, and all sample was purged with nitrogen gas prior to measurements.

Fluorescence microscopy imaging of differentiated human cholinergic SH-SY5Y cells.

The SH-SY5Y human neuroblastoma cell line is a cell model that is extensively used in neuroscience research. Given the ease of cultivation and quick differentiation into cholinergic neuronal cells, the SH-SY5Y human neuroblastoma cell line was selected as the cell model system for the *in vitro* detection of ACh release with the biosensor. Undifferentiated SH-SY5Y cells are widely reported to produce catecholamines.[8, 12] However, these cells can be differentiated into various neuronal subtypes capable of producing different neurotransmitters by addition of different growth factors at different time points.[6, 13, 14] The differentiation of SH-SY5Y cells with the use of RA and BDNF is a commonly used method of generating cholinergic SH-SY5Y cells.[14, 15] According to the extensive characterization of differentiated SH-SY5Y by de Medeiros and colleagues, the catecholaminergic phenotype was found to significantly decrease while cholinergic markers increased upon the completion of differentiation.[6]

The human cholinergic cells used in this study were differentiated from SH-SY5Y cells using the optimized protocol established by de Medeiros and colleagues for the detection of ACh exocytosis using the ultrafast chemically selective amperometric ACh biosensor (Figure S4).[6] Undifferentiated SH-SY5Y cells are fast-growing cells, but the differentiation protocol was found to slow down the rate of proliferation. Hence, the differentiated human cholinergic cells exhibited a slower proliferation rate and showed neuronal morphology with high neurite density and elongated neurites, as observed in Figure S4 A. Investigation of FM1-43 fluorescence in these cells showed that the cells were fully capable of exocytosis and endocytosis (Figure S4 B) and indicated an abundance of clear core synaptic-like vesicles presumably filled with ACh. The immunofluorescence data reported by Goodall and colleagues also demonstrated that neurites were rich in vesicles filled with neurotransmitters;[12] hence, axon terminals were selected as the major site of interest for exocytosis measurements rather than the cell body.

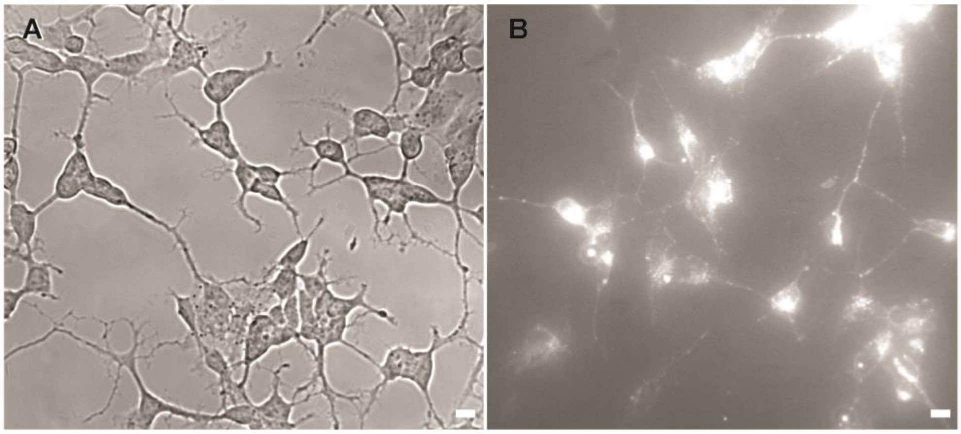

Figure S4. (A) Bright-field image of human cholinergic cells differentiated from SH-SY5Y cells at day 8. The cells appeared neuronal, with elongated neurites forming connections with adjacent cells. (B) Fluorescent image of differentiated human cholinergic SH-SY5Y cells labelled using FM1-43 dye. The scale bars in (A) and (B) represent 5 µm.

The presence of small clear core synaptic-like vesicles (SVs) in differentiated human cholinergic SH-SY5Y cells.
The electrochemical detection using a chemically selective ACh biosensor demonstrated the release of ACh from vesicles in differentiated human cholinergic SH-SY5Y cells. For estimation of the intravesicular ACh concentration, often referred to as the vesicle quantal size, confirmation of the vesicle dimensions of these cells is necessary. The presence of large dense core vesicles (LDCVs) containing norepinephrine and small synaptic-like vesicles without norepinephrine was previously reported by Goodall and colleagues in undifferentiated cholinergic SH-SY5Y cells using immunocytochemistry and immunofluorescence.[12] However, extensive characterization of SVs in SH-SY5Y cells, especially in differentiated human cholinergic SH-SY5Yc cells, appears to be lacking. ACh is reported to be found in SVs,[16, 17] which are located away from the soma and towards the axon terminals. The presence of SVs in differentiated human cholinergic SH-SY5Y cells was examined with transmission electron microscopy (TEM) following chemical fixation of the cells. In TEM micrographs of axons and the periphery of the differentiated cholinergic cells, both small SVs and LDCVs were observed as shown Figure 2 C. The average size of the SVs was estimated by measuring the horizontal and vertical diameter of individual vesicles in the TEM images using the Image J software. By averaging the horizontal and vertical vesicle measurements of the TEM photos, the vesicles in differentiated human cholinergic SH-SY5Y cells demonstrated a mean outer diameter of 46.2 nm ± 8.5 nm (standard deviation) or 46.2 nm ± 1.2 nm (standard error of the mean) (n = 55, at three different locations). Additional analysis using a Gaussian fitting to the histogram of the measured SV size distribution estimated the diameter of these vesicles to around 44 nm as shown in the equation (Figure S5). It is important to note that the sample size analyzed in this study may not be sufficient to determine the size distribution of the SVs with high accuracy. The main objective of this experiment is to confirm the presence of SVs, as previously reported in other studies,[18-21] rather than to determine their size. The observed SVs ranged from 40 to 60 nm, a size range widely accepted for this type of vesicle.[19]

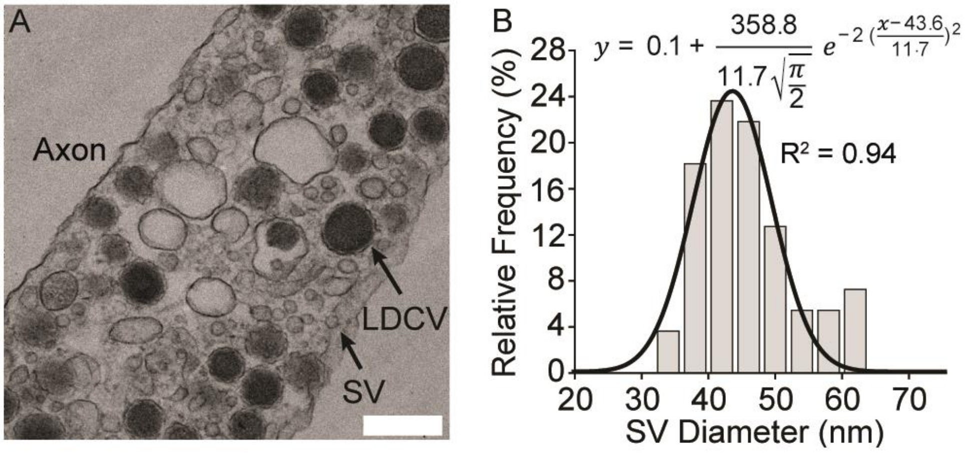

Figure S5. (A) Transmission electron microscopy image displaying the presence of small clear core synaptic-like vesicles (SV) and large dense core vesicles (LDCV) inside a differentiated cholinergic SH-SY5Y cell axon. Scale bar: 300 nm. (B)Histogram of small clear core vesicle size distribution with a bin size of 4 nm. It is analyzed from transmission electron microscopy micrographs.

Characterization of fusion pore controlled ACh quantal release suggests different modes of exocytosis.

After manual analysis of the individual spikes (n=2738) from exocytotic ACh release events at the axon terminals of differentiated human cholinergic cells (n=18), the amperometric spike parameters were summarized in Table S1. The typical fast current rise, slower current decay and a kinetic half-time on sub-millisecond time scale support the capability for the ACh sensor to temporally resolve single vesicle exocytosis events.

Table S1. Current spike analysis from amperometric recording of single exocytotic ACh release events at the axon terminals of differentiated human cholinergic cells (n=18). *

	T_{base} (ms)	$T_{1/2}$ (ms)	T_{rise} (ms)	T_{fall} (ms)	I_{max} (pA)	Q (fC)	Spike Number
Average (n = 18 cells)	1.4	0.5	0.3	0.3	6.3	4.1	2738
SEM	0.1	0.0	0.0	0.0	0.4	0.2	2738

* The data are presented as the average of means ± standard error of the mean (SEM).

By then sorting the individual recorded current spikes into groups according to the classified dynamic spike shapes, as illustrated in Figure 2D, the average values for the different kinetic and quantitative current spike parameters analyzed were plotted for each defined category of spike together with the spike shape prevalence in recordings (Figure S6). This analysis revealed a distinct variation in release kinetics for the different spike types, indicating that variable factors govern the fate of the fusion pore (dilation, flickering or closing) and the temporal dynamics after fusion pore formation. By controlling the fusion pore size and dynamics when open, the amount and speed of ACh release can be modulated (Figure S6 A-E). Therefore, the variability in fusion pore dynamics during the exocytosis events and variability on the amount acetylcholine released suggests different modes of exocytosis is present at these cells. This variability in dynamic release of ACh can potentially be attributed to the involvement of the SNARE-mediated exocytosis machinery, which governs the formation of the fusion pore and the biophysical nature where both lipids and proteins are thought to affect the fate of the fusion pore. Factors affecting the fusion pore machinery, such as the transient intracellular levels of calcium may play a crucial role in regulating neurotransmitter release and could play a role in the heterogeneity of spike shapes seen in our recordings.

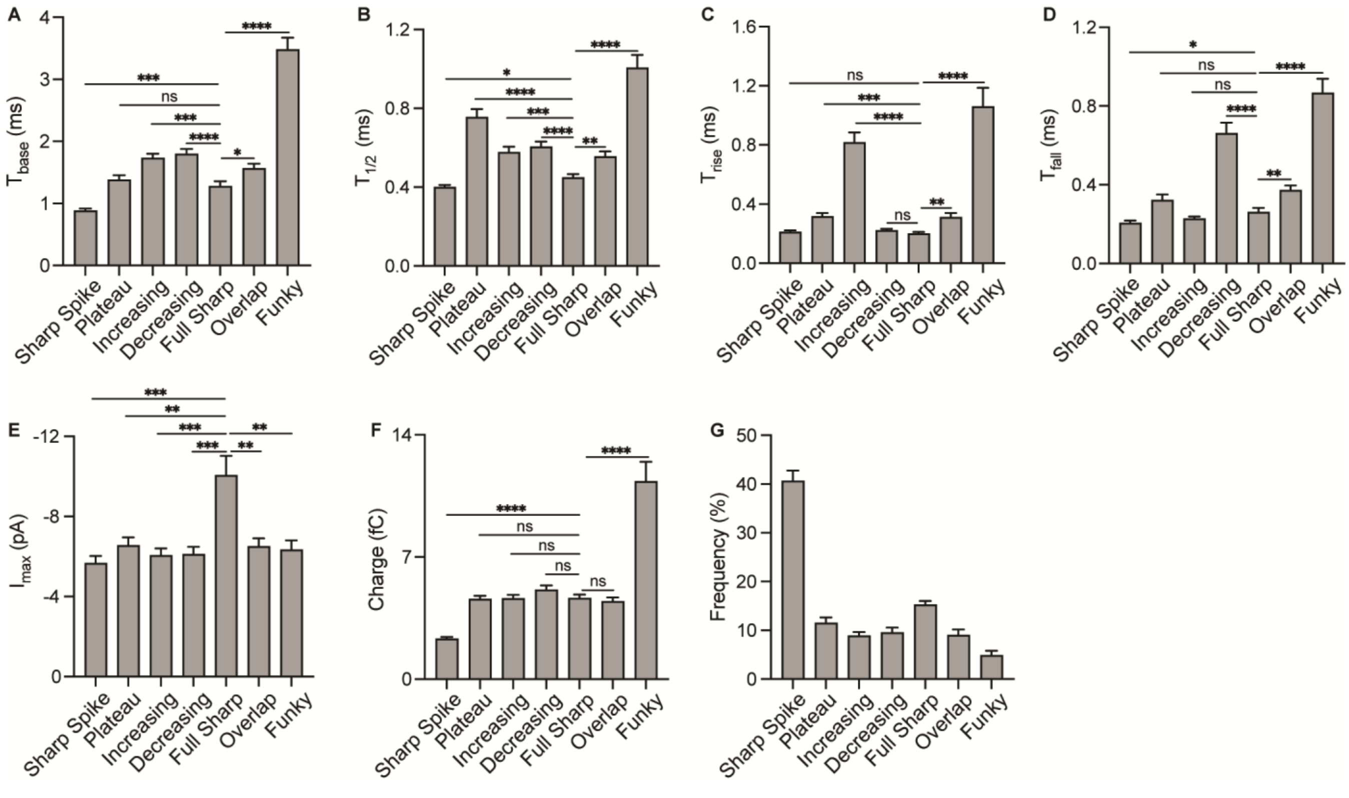

Figure S6. Plots of various analyzed amperometric reduction current spike parameters from recording of single exocytotic ACh release events from human differentiated cholinergic cells, for the seven different spike types. The four different kinetic parameters (A) spike base time, T_{base} (B) spike half-time, $T_{1/2}$ (C) spike rise time, T_{rise} (D) spike fall time, T_{fall} and the quantitative parameters (E) spike amplitude, I_{max} (F) total charge, Q, were binned according to one of the seven defined current spike shapes: sharp (n=18 cells, 897 spikes), plateau (n=18 cells, 300 spikes), increasing (n=18 cells, 229 spikes), decreasing (n=18 cells, 288 spikes), full sharp (n=18 cells, 305 spikes), overlap (n=18 cells, 415 spikes) and funky (n= 16 cells, 103 spikes). (G)

The frequency of ACh exocytosis activity (n=20 amperometric traces) versus the characterized current spike shapes: sharp (n=966 spikes), plateau (n=344 spikes), increasing (n=247 spikes), decreasing (n=324 spikes), full sharp (n=326 spikes), overlap (n=459 spikes) and funky (n=113 spikes) and measured from 20 different cells. The data shown in (A) – (G) is plotted as the average of means ± SEM. Two-tailed unpaired Student's *t*-test was conducted to compare full sharp spike type to all other spike types in graphs A-F, where ns = not significant, $**p \leq 0.01$, $***p \leq 0.001$, $****p \leq 0.0001$.

Analyzing the temporal and quantitative spike parameters as a function of current spike shape we first identified 6 spike shapes similar to those previously reported in literature.[22,23] When analyzing all spikes with sharp features, we observed the presence of two populations in terms of the magnitude of current spike amplitude: a smaller (sharp spike) and larger (full sharp). Since the large sharp spikes with the highest amplitude displayed the same total charge Q as increasing, decreasing and plateau spikes we named this large spike "full sharp". The full sharp spike presented here has previously not been characterized elsewhere, but potentially one can speculate that it might be a spike type that previously have been classified together with the smaller sharp spikes. Comparing the analysis of current spike parameters for the newly detected full sharp spikes with the other 6 spike shapes showed that the ACh release events significantly differed in terms of I_{max} where full sharp spikes were taller than all other type of spikes (Figure S6 E). Comparing the total charge detected, (Q), which relates to the total amount of ACh released during exocytosis, no statistically significant difference was observed between full sharp spikes and increasing, decreasing, plateau or peak overlap, whereas smaller sharp spikes were found to release only about 55% of the amount of ACh released during full sharp events and funky spikes about 250% of that of full sharp spikes (Figure S6 F). However, since the different spike types detected at these cholinergic cells differ greatly in shape, clearly a single temporal parameter is not sufficient to describe the significant kinetic differences in fusion pore dynamics. Therefore, commonly used temporal parameters such as the $T_{1/2}$ alone cannot describe the kinetics of spikes with more complex shapes. A great diversity in temporal parameters was observed between the spike types such as spike base time, T_{base} (Figure S6 A), half time, $T_{1/2}$, (Figure S6 B), rise time, T_{rise} (Figure S6 C) and fall time, T_{fall} (Figure S6 D). Figure S6 illustrates spikes of varying shapes and highlights how analysis of temporal spike parameters T_{base} and T_{half}, as well as I_{max} and Q reveals significantly different information of the spike types displays. In this figure the spikes have been extracted from a single amperometric recording and the graphs all use uniform time and current scale bars to show how spikes display distinct results of five representative spike types (Figure S7 A-E), where clearly the sharp spikes are significantly smaller and display different temporal parameters than the other spikes. While full sharp spike, increasing, decreasing, plateau might display similar T_{base}, $T_{1/2}$, and Q, it provides limited insight into the differences in their shape. Interestingly, the 6 spike shapes and their relative Q values are consistent with previously reported amperometric measurements of neurotransmitter systems such as glutamate signaling in rodent brain tissue and octopamine release in the brain of Drosophila melanogaster. [22, 23] Spikes with increasing, decreasing and plateau shapes were quantitatively identified as full exocytosis events, while sharp spikes were classified as partial exocytosis events. If the mechanisms observed in tissue also apply to cellular acetylcholine signaling, where broadly shaped spikes correspond to full exocytosis and sharp spike to partial exocytosis, this could suggest that full sharp spikes would correspond to full exocytosis.

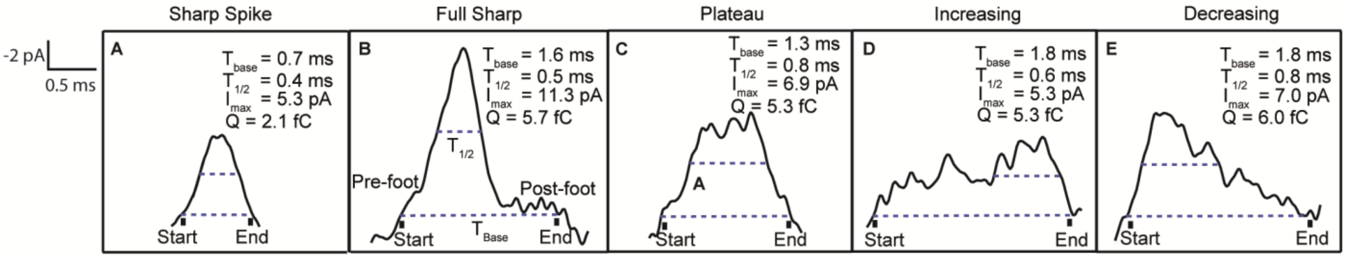

Figure S7. Single amperometric current spikes of ACh release when applying a -0.5 V potential to the ACh biosensor surface vs a Ag/AgCl reference electrode. The spikes include one partial release spike type: (A) sharp spike, and four distinct full release spike types: (B) full sharp, (C) plateau, (D) increasing, and (E) decreasing. All current spikes share the same current and time scale.

Additionally, by applying a high sampling rate of 50 kHz during the amperometric ACh exocytosis measurement at differentiated human cholinergic cells, the sharp current spikes that previously has been detected in other neurotransmitter systems at lower speed recording (5-10 kHz) and representing partial neurotransmitter release were further examined. The higher speed recording revealed that these rapid spikes also displayed intricate spike dynamics including various plateau shapes, skewed spikes (increasing or decreasing). Pre-spike and post-spike "foot" features that are commonly detected during partial release modes such as kiss-and-run exocytosis were also detected (Figure S8). Pre-spike feet are thought to represent neurotransmitter leakage through the initially formed 2 nm sized fusion pore before the pore dilates

to release a larger amount of neurotransmitter. The subsequent post-spike foot is thought to be neurotransmitters releasing through a narrow fusion pore when the pore is closing again and preparing for vesicle recapture. The presence of pre-spike and post-spike feet in sharp spikes provides further insights into the details of the SNARE complex mediating nanopore opening and closure that is controlled by the dynamics of the vesicle fusion pore.

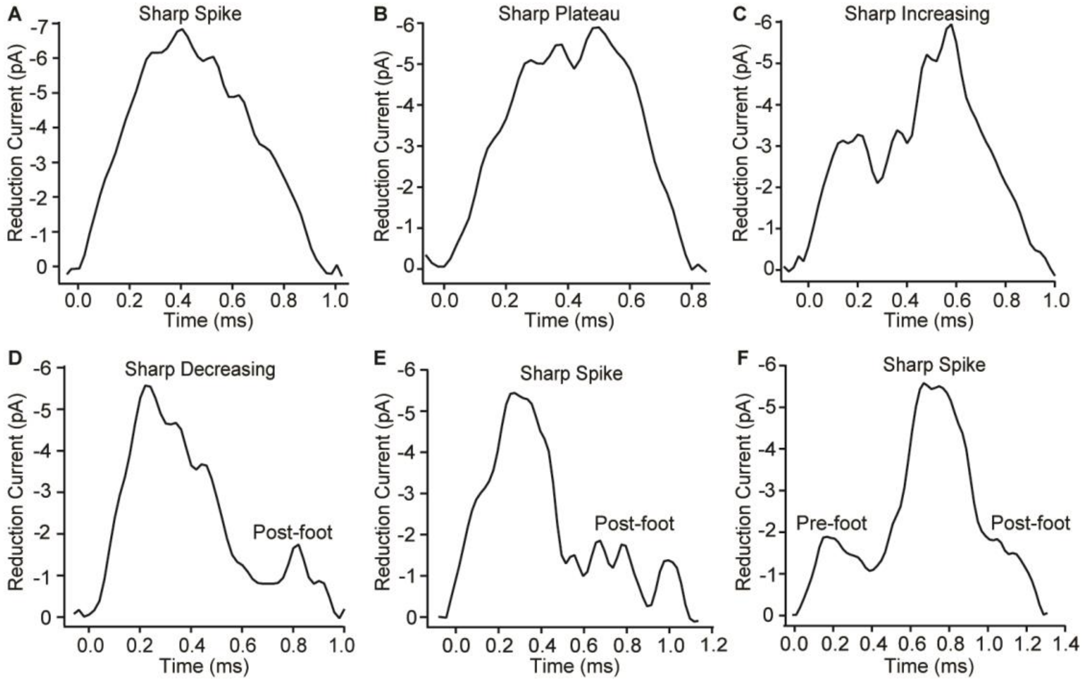

Figure S8. A display of the variation in complex current spike dynamics of "sharp" spikes detected in high temporal resolution amperometric recording of ACh release at differentiated cholinergic cells. These spikes that have been binned as "sharp" spikes because of their short time duration, low current amplitude and exhibit complex dynamics where representative spike features are presented as A) symmetric sharp spikes B) "plateau" sharp spikes C) a "sharp increasing" spike D) "sharp decreasing" spike with a post-foot E) "symmetric" sharp spike with a post-foot F) a "symmetric" sharp spike with a "pre-foot" and a "post-foot.

Bafilomycin A1 increases frequency and amount of quantal exocytosis release of ACh in differentiated human cholinergic cells.

High temporal amperometry recording can aid in the investigation of a drug's effects on neurotransmitter release at single cells since both alterations in exocytosis release probability and information on fusion pore-controlled changes in kinetic and quantitative neurotransmitter release during exocytosis can be obtained. Here, the vacuolar H^+-ATPase inhibitor, bafilomycin A1, was utilized to evaluate the potential of detecting measurable drug effects by the ultrafast ACh amperometric biosensor used in this study. Exocytosis recording at differentiated cholinergic SHSY5Y cells was first performed in the presence of 0.002% DMSO as a control measurement before subjecting the cells to a 0.1 μM bafilomycin A1 solution containing 0.002% DMSO. Using a glass pipette and a microinjection pump (Eppendorf FemtoJet), the bafilomycin A1 drug was continuously sprayed onto the target single cell for 10 minutes while simultaneously performing a continuous recording of exocytosis release of ACh at the cell exposed to the drug delivery (Figure 2). To minimize measurement variability from using different biosensors and different batches of cells, each pair of control and drug delivery measurements was performed using the same biosensor at the same location of the target cells. In the continuous amperometric recording of these cells, brief refractory periods were observed followed by long trains of exocytosis bursts. As shown in Figure 1G and H, the representative amperometric traces recorded in response to the administration of bafilomycin A1, the exocytosis activity drastically intensified with an increase in the frequency of exocytosis events by 160%.

Analysis of the data from the ACh exocytosis recordings at drug treated cells versus control was performed in terms of the amperometric current spike kinetic and quantitative parameters are summarized in Table S2. The data showed that after 10 minutes of drug administration, the average values for spike time duration (T_{base}), halftime ($T_{1/2}$) and current amplitude (I_{max}) were only slightly larger after drug treatment, whereas the quantal release was enhanced by 120 % compared to DMSO vehicle control. The compound effect is further illustrated by the histogram where the Gaussian

distribution of measured ACh quantal release is plotted before and after 10-minute drug administration (Figure S9). It demonstrates a shift of the distribution center to larger size after drug treatment, indicating a higher quantal release.

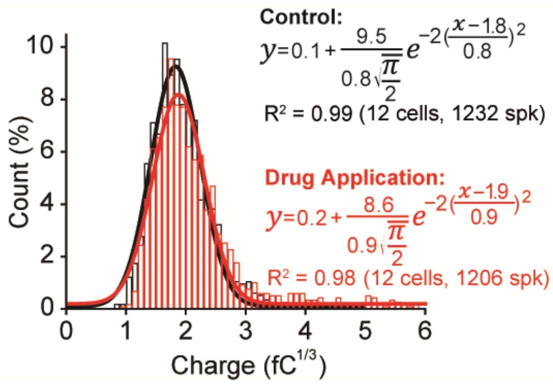

Figure S9. Histogram showing the distribution of quantal ACh exocytotic release from cholinergic differentiated SH-SY5Y cell before (black) and after 10-minute of Bafilomycin A1 administration (red) using amperometric ACh recording. For control experiments, cells were subjected to a bath of 0.002% DMSO, whereas for drug effect experiments, cells before and after 10-minute administration of 0.1 µM bafilomycin A1 application.

Table S2. Analysis of current spike kinetics, dynamics and frequency from amperometric recording of single ACh exocytosis events at the axon terminals of 13 differentiated human cholinergic cells before and after a 10-minute application of Bafilomycin A1.*

	T_{base} (ms)	$T_{1/2}$ (ms)	T_{rise} (ms)	T_{fall} (ms)	I_{max} (pA)	Q (fC)	Frequency	Spike No.
Control (n=13)	1.79 ± 0.13	0.66 ± 0.04	0.34 ± 0.03	0.37 ± 0.03	12.3 ± 1.7	9.7 ± 1.4	38 ± 8	1286
10 min after Bafilomycin A1 (n=13)	2.19 ± 0.23	0.71 ± 0.04	0.37 ± 0.03	0.47 ± 0.04	16.0± 3.9	15.8 ± 5.4	61 ± 13	1309

* The data are presented as the average of means ± SEM.

Measurement of LUV size and amperometric release of ACh from single ACh-filled LUVs.

The average diameter of single LUVs encapsulating different concentrations of ACh solution was measured using NTA, as shown in Figure S10A. The NTA data is presented as the mean ± standard error of the mean (SEM). The sizes of LUVs pre-filled ACh solution at different concentrations ranging from 200 mM to 600 mM were determined to be 183 ± 4 nm, 185 ± 1 nm, 180 ± 4 nm, 189 ± 8 nm, and 173 ± 3 nm, respectively. Figure S10B displays an averaged reduction current transient from 223 amperometric spikes of single LUVs bursting and releasing their content of a 400 mM ACh solution.

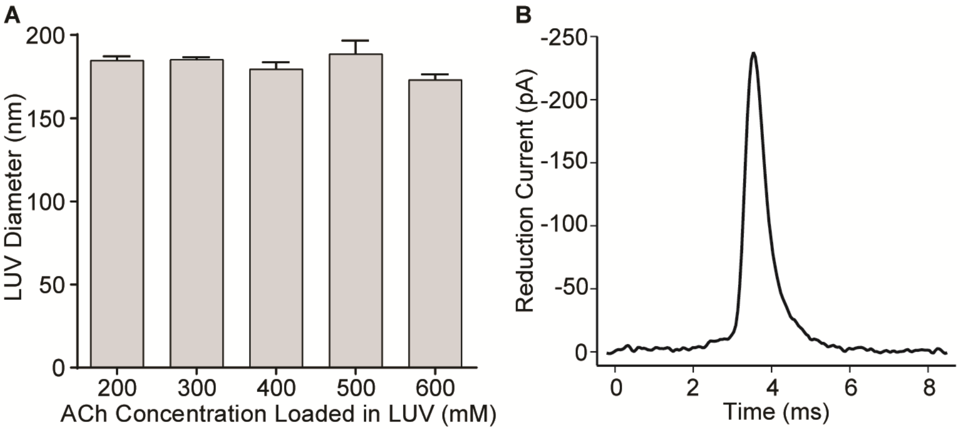

Figure S10. (A) The average LUV diameter at various concentrations of acetylcholine (ACh) solution encapsulated inside the LUVs (n = 3 ~ 5 LUV samples/LUV ACh concentration) as determined using NTA. The data is shown as mode ± standard error of the mean (SEM). (B) The averaged amperometric reduction current transients detected from ACh release of single LUV pre-filled with 400 mM ACh (n=223) bursting at the ACh biosensor surface. (The amperometry recording was performed by applying a -0.5 V *vs* a Ag/AgCl reference electrode.

Analysis of the dynamic and quantitative current spikes parameters from single LUV ACh release.
Table S3 summarizes the amperometric current spike parameters from the detection of ACh release from single LUVs filled with ACh that are bursting at the surface of the ACh biosensor. These current spikes represent ACh release from individual LUVs pre-filled with ACh solution, with concentrations varying from 200 mM to 600 mM. The analyzed current spike parameters included those associated with the kinetics of ACh release, such as the current spike half-width ($T_{1/2}$), rise time (T_{rise}) fall time (T_{fall}) total event time T_{base} and the maximum current spike amplitude (I_{max}) and additionally the total detected charge (Q) serving as a quantitative measure of the total amount ACh released from each rupturing LUV at the electrode surface. Although the quantitative parameters clearly increase with the increasing ACh concentration inside the LUVs, the kinetic parameters showed non-significant difference suggesting that the mechanism for ACh release was not affected by the ACh concentration inside the LUVs.

Table S3. Amperometric current spike parameters obtained via bursting of single LUVs pre-filled with various concentrations of ACh solution ranging from 200 mM to 600 mM.*

ACh Concentration loaded in LUV (mM)	T_{base} (ms)	$T_{1/2}$ (ms)	T_{rise} (ms)	T_{fall} (ms)	I_{max} (pA)	Q (fC)	Replicate #	Sensor #	Spike #	Trace #
200	1.89 ± 0.27	0.60 ± 0.07	0.27 ± 0.03	0.39 ± 0.06	144 ± 26	101 ± 15	5	6	2139	19
300	2.53 ± 0.64	0.60 ± 0.08	0.26 ± 0.03	0.40 ± 0.10	175 ± 36	131 ± 18	4	4	584	4
400	1.91 ± 0.15	0.61 ± 0.03	0.25 ± 0.01	0.33 ± 0.02	343 ± 33	225 ± 18	4	6	1751	15
500	1.93 ± 0.21	0.69 ± 0.09	0.25 ± 0.02	0.42 ± 0.09	337 ± 45	280 ± 75	4	5	1067	11
600	2.27 ± 0.44	0.56 ± 0.10	0.24 ± 0.05	0.40 ± 0.07	506 ± 130	403 ± 88	5	9	2434	20

* The data are presented as the average of means ± SEM.

A calibration curve for charge density versus ACh concentration in pre-filled LUVs can be plotted for calibrating the ACh biosensors as previously described.[1] In brief, the charge densities of each concentration of ACh were calculated by dividing the average current spike integrated charge Q from amperometry experiments by the inner volume of LUVs as measured using NTA. This calibration curve then allows to quantify the intravesicular ACh content in the amperometric recordings from human cholinergic cells.

In summary, the amperometric data presented here demonstrate the successful fabrication of our newly developed ACh biosensor, primed for ultrafast detection of ACh release from cells. Utilizing the biosensor calibration with ACh-filled LUVs (Figure 2C), our experiments enable precise quantitative measurements of ACh release from fusion pore-regulated exocytosis events at differentiated human cholinergic SH-SY5Y cells.

III. References

(1) Wang, Y. M.; Fathali, H.; Mishra, D.; Olsson, T.; Keighron, J. D.; Skibicka, K. P.; Cans, A. S. Counting the Number of Glutamate Molecules in Single Synaptic Vesicles. J Am Chem Soc 2019, 141 (44), 17507-17511. DOI: 10.1021/jacs.9b09414.

(2) Wang, Y.; Pradhan, A.; Gupta, P.; Karlsson-Fernberg, H.; Cans, A.-S. Quantitative Analysis of Single Glutamatergic Vesicles in the Brain. In New Technologies for Glutamate Interaction: Neurons and Glia, Kukley, M. Ed.; Springer US, 2024; pp 91-120. DOI:10.1007/978-1-0716-3742-5_5

(3) Keighron, J. D.; Wigstrom, J.; Kurczy, M. E.; Bergman, J.; Wang, Y. M.; Canst, A. S. Amperometric Detection of Single Vesicle Acetylcholine Release Events from an Artificial Cell. Acs Chemical Neuroscience 2015, 6 (1), 181-188. DOI: 10.1021/cn5002667.

(4) Finot, M. O.; Braybrook, G. D.; McDermott, M. T. Characterization of electrochemically deposited gold nanocrystals on glassy carbon electrodes. J Electroanal Chem 1999, 466 (2), 234-241. DOI: Doi 10.1016/S0022-0728(99)00154-0.

(5) Keighron, J. D.; Akesson, S.; Cans, A. S. Coimmobilization of Acetylcholinesterase and Choline Oxidase on Gold Nanoparticles: Stoichiometry, Activity, and Reaction Efficiency. Langmuir 2014, 30 (38), 11348-11355. DOI: 10.1021/la502538h.

(6) de Medeiros, L. M.; De Bastiani, M. A.; Rico, E. P.; Schonhofen, P.; Pfaffenseller, B.; Wollenhaupt-Aguiar, B.; Grun, L.; Barbe-Tuana, F.; Zimmer, E. R.; Castro, M. A. A.; et al. Cholinergic Differentiation of Human Neuroblastoma SH-SY5Y Cell Line and Its Potential Use as an In vitro Model for Alzheimer's Disease Studies. Mol Neurobiol 2019, 56 (11), 7355-7367. DOI: 10.1007/s12035-019-1605-3.

(7) Betz, W. J.; Bewick, G. S. Optical Analysis of Synaptic Vesicle Recycling at the Frog Neuromuscular-Junction. Science 1992, 255 (5041), 200-203. DOI:10.1126/science.1553547.

(8) Lazarenko, R. M.; DelBove, C. E.; Zhang, Q. Fluorescent Measurement of Synaptic Activity Using FM Dyes in Dissociated Hippocampal Cultured Neurons. Bio-Protocol 2018, 8 (2). DOI: ARTN e269010.21769/BioProtoc.2690.

(9) Costa, A. P.; Xu, X. M.; Burgess, D. J. Freeze-Anneal-Thaw Cycling of Unilamellar Liposomes: Effect on Encapsulation Efficiency. Pharm Res-Dordr 2014, 31 (1), 97-103. DOI: 10.1007/s11095-013-1135-z.

(10) Mosharov, E. V.; Sulzer, D. Analysis of exocytotic events recorded by amperometry. Nat Methods 2005, 2 (9), 651-658. DOI:10.1038/nmeth782.

(11) Schneider, C. A.; Rasband, W. S.; Eliceiri, K. W. NIH Image to ImageJ: 25 years of image analysis. Nat Methods 2012, 9 (7), 671-675. DOI: 10.1038/nmeth.2089.

(12) Goodall, A. R.; Danks, K.; Walker, J. H.; Ball, S. G.; Vaughan, P. F. Occurrence of two types of secretory vesicles in the human neuroblastoma SH-SY5Y. J Neurochem 1997, 68 (4), 1542-1552. DOI: 10.1046/j.1471-4159.1997.68041542.x

(13) Encinas, M.; Iglesias, M.; Liu, Y.; Wang, H.; Muhaisen, A.; Cena, V.; Gallego, C.; Comella, J. X. Sequential treatment of SH-SY5Y cells with retinoic acid and brain-derived neurotrophic factor gives rise to fully differentiated, neurotrophic factor-dependent, human neuron-like cells. J Neurochem 2000, 75 (3), 991-1003. DOI: 10.1046/j.1471-4159.2000.0750991.x

(14) Kovalevich, J.; Langford, D. Considerations for the use of SH-SY5Y neuroblastoma cells in neurobiology. Methods Mol Biol 2013, 1078, 9-21. DOI: 10.1007/978-1-62703-640-5_2

(15) Xicoy, H.; Wieringa, B.; Martens, G. J. The SH-SY5Y cell line in Parkinson's disease research: a systematic review. Mol Neurodegener 2017, 12 (1), 10. DOI: 10.1186/s13024-017-0149-0

(16) Bauerfeind, R.; Regnier-Vigouroux, A.; Flatmark, T.; Huttner, W. B. Selective storage of acetylcholine, but not catecholamines, in neuroendocrine synaptic-like microvesicles of early endosomal origin. Neuron 1993, 11 (1), 105-121. DOI: 10.1016/0896-6273(93)90275-v

(17) Westfall, T. C. Cholinergic Neurotransmission in the Autonomic and Somatic Motor Nervous System. In Encyclopedia of Neuroscience, Squire, L. R. Ed.; Academic Press}, 2009; pp 827-834. DOI: 10.1016/B978-008045046-9.01133-5

(18) Harris, K. M.; Sultan, P. Variation in the Number, Location and Size of Synaptic Vesicles Provides an Anatomical Basis for the Nonuniform Probability of Release at Hippocampal Ca1 Synapses. Neuropharmacology 1995, 34 (11), 1387-1395. DOI: Doi 10.1016/0028-3908(95)00142-S.

(19) Purves, D.; Augustine, G. J.; Fitzpatrick, D.; Katz, L. C.; LaMantia, A.-S.; McNamara, J. O. & Williams, S. M. (2001). Neuroscience. 2nd Edition (2nd ed.). Sinauer Associates. https://www.ncbi.nlm.nih.gov/books/NBK11166/

(20) Qu, L.; Akbergenova, Y.; Hu, Y. M.; Schikorski, T. Synapse-to-Synapse Variation in Mean Synaptic Vesicle Size and Its Relationship With Synaptic Morphology and Function. J Comp Neurol 2009, 514 (4), 343-352. DOI: 10.1002/cne.22007.

(21) Takamori, S.; Holt, M.; Stenius, K.; Lemke, E. A.; Gronborg, M.; Riedel, D.; Urlaub, H.; Schenck, S.; Brugger, B.; Ringler, P.; et al. Molecular anatomy of a trafficking organelle. Cell 2006, 127 (4), 831-846. DOI: 10.1016/j.cell.2006.10.030.

(22) Majdi, S.; Berglund, E. C.; Dunevall, J.; Oleinick, A. I.; Amatore, C.; Krantz, D. E.; Ewing, A. G. Electrochemical Measurements of Optogenetically Stimulated Quantal Amine Release from Single Nerve Cell Varicosities in Larvae. Angew Chem Int Edit 2015, 54 (46), 13609-13612. DOI: 10.1002/anie.201506743.
(23) Wang, Y. M.; Mishra, D.; Bergman, J.; Keighron, J. D.; Skibicka, K. P.; Cans, A. S. Ultrafast Glutamate Biosensor Recordings in Brain Slices Reveal Complex Single Exocytosis Transients. Acs Chemical Neuroscience 2019, 10 (3), 1744-1752. DOI: 10.1021/acschemneuro.8b00624.
